# Circuit to Construct Mapping: A Mathematical Tool for Assisting the Diagnosis and Treatment in Major Depressive Disorder

**DOI:** 10.3389/fpsyt.2015.00029

**Published:** 2015-02-26

**Authors:** Natalia Z. Bielczyk, Jan K. Buitelaar, Jeffrey C. Glennon, Paul H. E. Tiesinga

**Affiliations:** ^1^Donders Institute for Brain, Cognition and Behavior, Nijmegen, Netherlands; ^2^Department of Cognitive Neuroscience, Radboud University Nijmegen Medical Centre, Nijmegen, Netherlands; ^3^Department of Neuroinformatics, Radboud University Nijmegen, Nijmegen, Netherlands

**Keywords:** major depressive disorder, modeling, circuit, diagnosis, research domain criteria project, dynamical systems

## Abstract

Major depressive disorder (MDD) is a serious condition with a lifetime prevalence exceeding 16% worldwide. MDD is a heterogeneous disorder that involves multiple behavioral symptoms on the one hand and multiple neuronal circuits on the other hand. In this review, we integrate the literature on cognitive and physiological biomarkers of MDD with the insights derived from mathematical models of brain networks, especially models that can be used for fMRI datasets. We refer to the recent NIH research domain criteria initiative, in which a concept of “constructs” as functional units of mental disorders is introduced. Constructs are biomarkers present at multiple levels of brain functioning – cognition, genetics, brain anatomy, and neurophysiology. In this review, we propose a new approach which we called circuit to construct mapping (CCM), which aims to characterize causal relations between the underlying network dynamics (as the cause) and the constructs referring to the clinical symptoms of MDD (as the effect). CCM involves extracting diagnostic categories from behavioral data, linking circuits that are causal to these categories with use of clinical neuroimaging data, and modeling the dynamics of the emerging circuits with attractor dynamics in order to provide new, neuroimaging-related biomarkers for MDD. The CCM approach optimizes the clinical diagnosis and patient stratification. It also addresses the recent demand for linking circuits to behavior, and provides a new insight into clinical treatment by investigating the dynamics of neuronal circuits underneath cognitive dimensions of MDD. CCM can serve as a new regime toward personalized medicine, assisting the diagnosis and treatment of MDD.

## Introduction

### Major depressive disorder

Major depressive disorder (MDD), also known as unipolar depression, has a lifetime prevalence that exceeds 16% in the US (1), and is expected to increase their share in the global disease burden from 4.3% in 2004 to 6.2% by 2030 (2). Treating MDD is costly. In 2010, the total cost of MDD in the EU was estimated to be €798 billion, of which 60% was direct costs and 40% due to lost productivity (3). Currently, there is a rich variety of competing biomarker sets, each suggesting different MDD etiology. However, it is unclear how these relate to the current diagnostic criteria. This heterogeneity of biomarkers, behavioral symptoms, and circuit changes in MDD requires the use of multimodal and multidisciplinary approaches together with mathematical modeling in order to integrate these findings into diagnostic and intervention tools useful in clinical practice.

So far, the search for candidate genes underlying MDD has not yielded a single responsible gene. Instead, genetic models of MDD propose that a large number of genes is involved (4), with a small contribution of each of them to MDD phenotype. Furthermore, these models suggest that epigenetic regulation may underlie critical gene-environment effects in MDD (5). Epidemiological studies have revealed that genetic factors may account for 40–50% of the risk of developing the disorder (6). Since the definition of an endophenotype involves heritability (7) and can only be used in a family sensitive design (8), it leads to a conclusion that only particular diagnostic categories in MDD can be interpreted as endophenotypes. Therefore, instead of talking about endophenotypes in MDD, we refer to NIH research domain criteria (RDoC) project approach (9) and to its central concept of a *construct* as a basic dimension of brain functioning (without a requirement of heritability). While defining constructs, RDoC initiative refers to various units of analysis, from genes to neural circuits and behavior.

In section “Etiology of MDD”, we review the current state of knowledge about MDD etiology across multiple construct domains, from behavioral through physiological down to neuronal level. Furthermore, we propose a new paradigm to aid in the diagnosis of MDD and its clinical management which includes dynamical models of the underlying circuitry and mapping the activity of these circuits onto cognitive constructs diagnostic for MDD. This circuit to construct mapping (CCM) approach can facilitate a personalized approach to MDD and thereby improve the quality of life for MDD patients.

### Causality

Mapping the activity of underlying circuits onto cognitive constructs diagnostic for MDD involves assumption that we can point to causal relations between these two domains. In this review, we focus on the altered dynamics of neuronal circuits as the cause of disrupted behavior. But how can one determine causality? There are two definitions of causality, and both of which are often used in research. First definition by Lewis (10) describes causality in the language of *counterfactuals*: we may define a cause to be an object followed by another, where, if the first object had not been, the second never had existed. On the basis of this definition, in 1986, Holland formulated the “no causation without manipulation” rule (11) which became the prevailing principle in causal research for another two decades. Today, Woodward’s view at causality through structural equations comes popular (12). Assuming that we have an endogenous variable *Y*, produced from variables *X*_1_, *X*_2_, …, *X*_n_, Woodard’s approach involves expressing certain basic counterfactuals in the following form: *If it were the case that X*_1_ = *x*_1_*, X*_2_ = *x*_2_, …, *X_n_* = *x_n_, then it would be the case that Y* = *f* (*x*_1_, …, *x_n_*).

However, this is not the only view on causality. Judea Pearl builds in the counterfactual approach and writes in his recent essays (13): “the essential ingredient of causation is responsiveness, namely, the capacity of some variables to respond to variations in other variables, regardless of how those variations came about.” This is an objection to the idea that the establishment of causation necessarily requires manipulation; rather, it is sufficient to observe the system and its natural course. However, the inference of causality on the basis of observational data is not easy, and Pearl developed a comprehensive theory of how to establish causation by means of probabilistic models.

This latter view of causality is beneficial to causal research in psychiatry; because, we are not always equipped with tools to manipulate all the candidate causes in our system. For instance, if we are interested in the causal effect of the insular cortex on emotional states in patients with MDD and we aim to apply the counterfactual approach in order to test this hypothesis, we should shut down the activity of the isolated insula and register the observed change in regulation of emotional states in our cohort. However, since the insula does not lay on the surface of the cortex, it is very hard to non-invasively perturb its activity alone; since, so far the remote control of deep brain activity is not available in humans. Therefore, in clinical trials the second definition of causality is typically applied: one compares a population of subjects with and without overactivation in the insular cortex, and tries to find systematic differences between these two groups in terms of emotional states. If the effect size is large enough for the groups of a given amount of patients, the causal effect is determined. In the further sections, we will discuss causality in Pearl’s sense, meaning “observation” and “statistical power” rather than “intervention” and “counterfactuals.”

## Etiology of MDD

### Constructs in MDD

Causality in case of MDD (and other cognitive disorders) is a complex research problem because the disorder can be described across various domains, from neurophysiology, through neuronal networks, to behavior. Although a causal explanation in MDD can search for relationships between any pair of constructs, from the psychiatric point of view links in which behavioral constructs are the effect are especially valuable.

**Figure 1:**
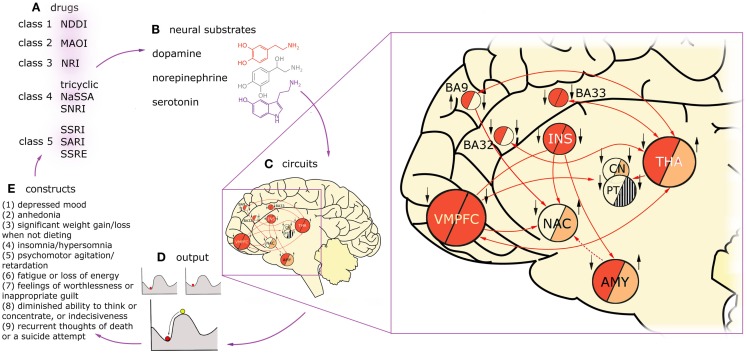
**Diagnosis, treatment, and brain dynamics in MDD**. One can distinguish five classes of drugs **(A)** on the basis of monoamine receptors that they target **(B)**. Functional MRI studies reveal that these drugs affect different, but overlapping circuits **(C)**. Here, two exemplary circuits are presented on the basis of imaging studies on two constructs present in MDD: low mood (or negative affect) and anhedonia (lack of positive affect). The connectivity in the circuits is presented with arrows, solid lines for glutamatergic, and dashed lines for GABAergic projections. Findings on regions activated in negative affect in depressed patients are summarized in the “negative affect” circuit, left half-circles. The subjects were triggered to fall into low mood by presenting them with scenes of negative emotional valence. Findings on regions up- and down-regulated in anhedonic MDD patients during presentation of scenes with positive emotional valence are summarized in the “anhedonia” circuit, right half-circles. Deep red color depicts overactivation in a given region during the task in respect to healthy controls, light red – hypoactivation, stripes – counteracting evidence in the literature, background color – no data. Additionally, treatment effects of fluoxetine in the nodes of this network are indicated with black arrows (up: up-regulation in respect to non-medicated patients; down: down-regulation). Influence of the drug was assessed on the basis of imaging studies that were using experimental tasks focusing on emotion processing (as aforementioned tasks involving presentation with scenes of emotional valence). On the left hand side of each region, influence of fluoxetine treatment in negative affect regime in MDD is indicated, on the right hand side: the same for positive affect regime. On one hand, this figure demonstrates that drugs act on constructs rather than particular brain regions. On the other hand, it shows that circuits underlying constructs are strongly overlapping but not identical. For any given construct, the underlying circuitry, modulated by interplay between the neural substrates within, flows toward a stable activity pattern – the attractor state **(D)**. The stability means the network will relax to the same stable pattern even after small degree of external stimulation. This phenomenon is pictured with the ball metaphor. Treatment with drugs is most likely to change the patient’s state by reshaping the attractor landscape. This drift results in change in the particular construct, whose circuit is targeted with treatment **(E)**, but may also affect other constructs via circuits overlapping with the targeted one. Then, the behavioral consequences of the treatment are the basis to prescribe a more appropriate drug for the given individual **(A)**. VMPFC, ventromedial prefrontal cortex, including BA25 (subgenual cortex); BA9, dorsolateral prefrontal cortex; BA32, dorsal anterior cingulate cortex; BA33, part of anterior cingulate cortex; INS, insula; NAC, nucleus accumbens; CN, caudate nucleus; PT, putamen; THA, thalamus; AMY, amygdala.

Figure 1 presents the variety of constructs across multiple levels of description in a process of a typical treatment in MDD, with arrows denoting causal relations between them. Firstly, one can distinguish five classes of drugs (Figure 1A) on the basis of monoamine receptors that they target (Figure 1B). A patient diagnosed with MDD is typically prescribed with one or, rarely, with a combination of these drug types. Functional MRI studies reveal that these drugs affect different, but overlapping circuits (Figure 1C). For any given construct, the underlying neuronal circuitry, modulated by interplay between the neural substrates within, reaches a stable activity pattern – which is pictured with the ball metaphor (Figure 1D). The network specific activation pattern, as we believe, modulates the particular cognitive construct (Figure 1E). The behavior of the patient is subject to repetitive diagnoses which, possibly, can lead to prescription of new, more accurate drugs which closes the circle. In our understanding, the mechanism underlying MDD is a superposition of multiple circuits, each of them having a causal effect on one of the cognitive constructs present in MDD. Therefore, in our considerations on modeling MDD, we are interested in the causal effect between neuronal circuits (as the cause, C) and behavioral constructs (as the effect, D).

We briefly review the aforementioned levels of the description in the following sections. Although the proposed CCM approach includes only mapping from neuronal circuitries straight to the cognitive domain, the physiology underlying MDD is also worth mentioning; because, the most popular (but not necessarily the most effective) treatments derive from the monoamine theory of MDD and target neuromodulatory receptors in the brain rather than particular circuits.

### Cognitive constructs

Major depression was originally defined in terms of behavior; therefore, cognitive constructs present in MDD seem to be the right starting point to give full characteristics of this disorder. In DSM-5, diagnostic criteria for MDD are as follows: if the subject is diagnosed with MDD if at least five out of nine diagnostic traits are present (Figure 1E), at least one of them being anhedonia or low mood.

Current diagnostic practice for MDD is difficult. First, both DSM-5 and ICD-10 diagnostic criteria allow for a broad range of behavioral profiles, all diagnosed with the same clinical condition (14, 15). Second, the diagnostic criteria are open to different interpretations, change over time and are therefore less objective and require review by trained clinicians. For example, independent symptoms of dysthymia (present in DSM-4 as a self-standing disorder) were recently classified as chronic MDD in DSM-5, because since DSM-4 was released there was not enough evidence that dysthymia is significantly different from MDD (16). Third, sometimes new MDD types are distinguished on the basis of specific events triggering the disorder, e.g., grief in the DSM-5 [and in the incoming ICD-11 (14, 17, 18)] and premenstrual dysphoric disorder in DSM-5 (19). This change of diagnostic criteria over time leads to differences in interpretation and is a strong argument for developing an objective approach.

### Physiological constructs

As mentioned before, there is a variety of competing biomarker sets, each suggesting different MDD etiology. The catecholamine hypothesis of Schildkraut (20), originated in the 60s, advocated that norepinephrine (NE) plays a pivotal role in affective disorders, with a lesser role for epinephrine (E), dopamine (DA), and serotonin (5HT) levels. The hypothesis suggested a reduced level of neurotransmission in E, NE, DA, and 5HT pathways as a possible cause of MDD. Today, it is known that not only DA, NE, and 5HT, but also acetylcholine (AC) has a strong impact on mood (21). Nevertheless, the mechanism of the shift from a healthy brain state into MDD and the role of each of these neuromodulators in this process are not yet understood.

Monoamines and AC are not the only neuromodulatory chemicals involved in MDD. Neuroendocrine mechanisms such as the corticotropin-releasing factor (CRF) may also play a role (22). In depression, this peptide is overproduced in the hypothalamus, which, acting along with arginine vasopressin (AVP), triggers hypersecretion of adrenocorticotrophic hormone (ACTH) from the pituitary. Overproduction of ACTH leads in turn to overproduction of glucocorticoids (cortisol in humans, corticosterone in rodents) from the adrenal cortex. This circuit is known as the hypothalamic-pituitary-adrenal (HPA) axis, and – as a part of the neuroendocrine system – it controls stress reactions, metabolism, and immunity (23). HPA theory of depression corresponds to the evidence that, due to epigenetic mechanisms, early life events can cause HPA overactivation in adult life (24).

Furthermore, recent observations demonstrate that antidepressant drugs targeting monoamines also modulate synaptic GABA transmission. Additionally, post-mortem studies reveal a dramatic reduction in plasmic GABA concentration in MDD patients. These findings have implicated GABAergic mechanisms in MDD (25), and led to the postulate that the balance of excitation and inhibition (E-I) in brain networks in MDD is disturbed (26).

Another theory of MDD results from the observation that antidepressants induce plasticity in the synaptic strengths, altering patterns of connectivity in the brain (27). Consequently, it was proposed that MDD may reflect a primary impairment in neuronal information processing caused by a disrupted functional or effective (directed) connectivity rather than by any form of chemical imbalance.

### Neuronal constructs

The identification of neuronal circuits underlying MDD with use of fMRI initially has led to the default mode network (DMN) theory of MDD (28, 29). DMN is a circuit defined by slow, coherent oscillatory activity in a wakeful resting state in humans with eyes closed (30). It mostly involves structures engaged in self-referential processes (parts of the medial prefrontal, posterior cingulate and parietal cortices, and medial temporal lobe), as well as the centers for memory (hippocampus, parahippocampal gyrus) and limbic structures (amygdala, nucleus accumbens, hypothalamus) (31). Imaging studies reveal that resting-state activity in many of the DMN nodes is altered in MDD (32). It was recently found that activity in DMN correlates with mood (33), therefore this circuit might be responsible for the affective aspect of the disorder. DMN is just one of many resting-state networks (RSNs) identified so far (34), and methods proposed for identification of MDD on the basis of resting state fMRI respect not only DMN but also other RSNs. For instance, a recently developed computational diagnostic method utilizing Hurst exponent takes into account DMN, right and left fronto-parietal, ventromedial prefrontal, and salience networks (35).

Recent evidence suggests that not only RSNs, but also the central-executive network (CEN) seems to be impaired in MDD (36). This network involves a few subdivisions of prefrontal cortex (PFC), anterior thalamus, and dorsal caudate nucleus. As opposed to RSNs, CEN comes to play during processing that requires cognitive control (37), and therefore is responsible for the executive functions, e.g., response inhibition, reward processing, planning, and working memory. Therefore, as opposed to RSNs, CEN might be involved in such constructs as recurrent thoughts of death and diminished attention. These two families of networks are complimentary and tend to switch the activity between each other.

Identification of common patterns of up- and down-regulation in the nodes of RSNs and CEN could serve as a new, more robust mean to identify network-related biomarkers of MDD (38). In particular, construct-based approach would allow for creating of individual dynamical profiles for patients, and therefore personalized therapy.

## Treatment

Coming back to causality, we believe that treatments in MDD affect neuronal dynamics, and this dynamics in turn triggers the behavioral change. Treatment choice depends on multiple factors, including the course of the disease, prior medical treatment, etc (39). Evidence-based treatment guidelines suggest cognitive-based therapy [CBT (40)] and pharmacology (41) as the first treatment of choice (42). On the other hand, electroconvulsive therapy [ECT (43)] is only recommended if the aforementioned methods are ineffective for the given patient, whereas deep brain stimulation [DBS (43)], as the most invasive method, is not yet approved by the United States Food and Drug Administration for treatment-resistant depression (43). Even though new treatment methods such as repetitive transcranial magnetic resonance [rTMS, a localized, superficial stimulation of the cortex with magnets (44)] and neurofeedback therapy [a combination of cognitive therapy with neurobiological approach: a real-time feedback of local fMRI signals (45)] are being tested, they are not established methods yet.

An example of drugs as a treatment procedure affecting construct-related circuits, changing the brain dynamical state, and thus influencing the diagnosis is presented in Figure 1A.

## Circuit for MDD

As mentioned in section “Constructs in MDD”, our viewpoint is that the mechanism underlying MDD is a superposition of multiple circuits, each of them having a causal effect on one of the cognitive constructs present in MDD. In fact, the number of these cognitive constructs, and therefore also the underlying circuits, may be much higher than the number of diagnostic categories specified in the DSM-5. Exemplary constructs not mentioned in the DSM-5 but present in a vast majority of MDD patients include negative bias in attention and memory (46), a negative view of the world and the future (41), learned helplessness (47), obsessions, and pathological rumination (48).

However, in order to perform a causal inference linking circuits to cognitive constructs, one needs to determine which circuits to study in the first place. MDD is a heterogenous disorder, and, as such, arises from anatomical and functional changes in a wide range of brain regions. The circuits that were first proposed to be responsible for MDD consisted of regions known to be involved in mood. One of these mood generators is the corticomesolimbic loop: one of a few parallel, basal ganglia-thalamo-cortical loops that projects from the ventromedial PFC to the medial dorsal thalamus through the nuclei of the basal ganglia (49). The other mood generator is the aforementioned hypothalamic-pituitary-adrenal axis (HPA) whose dysfunction widely affects monoamine pathways and triggers mood fluctuations. Recently, the viewpoint at MDD and other mental disabilities through the prism of large-scale brain networks identified on the basis of fMRI studies (RSNs and subcircuits of the CEN), and interactions between them, has gained in popularity (50–56).

We take this large-scale perspective. However, as mentioned above, in our view the search for mechanisms underlying MDD should include zooming into circuits underlying single diagnostic constructs. Large-scale networks are complex and, as such, they might be decomposed into simpler functional circuits. This is definitely the case for the CEN. On one hand, various cognitive constructs could be characterized as different states within the same network. On the other hand, CEN is most probably divided into functional subcircuits which activate while solving particular tasks involving cognitive control, e.g., reward receipt, signal inhibition, decision making, language processing. Another example is the DMN which generates mood. It might be composed of a few interacting subcircuits accounting for generation of basic emotions (57, 58) which do not coexist (59, 60). However, it could also be the case that basic emotions represent various attractors of one large circuit, which is why it is so hard to find specific neuronal underpinnings of basic emotions (61, 62).

In terms of models, so far RSNs are better characterized than CEN (63, 64), probably because of stable temporal dynamics that can be easily investigated with fMRI. Interestingly, Deco et al. (65) propose a model of the resting-state oscillations as a multistable system driven by noise, which is consistent with recent findings on the dynamics of the functional connectivity in RSNs (66–68). It turns out that resting state activity is not uniform but involves numerous modes that switch on and off. Some computational studies suggest that the identified modes of functional connectivity correspond to various eigenmodes of the anatomical connectivity (69), which is a strong argument toward a viewpoint at DMN and other RSNs as a number of interconnected circuits. On the contrary, psychometric studies reveal seven dimensions of cognition during rest: discontinuity of mind, theory of mind, self, planning, sleepiness, comfort, and somatic awareness (70). These dimensions represent various cognitive modes between which subjects switch during the rest. This is an argument on behalf of switching between attractors of one big network during the resting state.

How do the circuits generating single cognitive constructs contribute to this large-scale picture? The construct-wise approach that we take is motivated by circumstantial evidence that, in general, drugs target cognitive constructs rather than the whole disorders. Figure 1C presents an example of fluoxetine acting differently in MDD patients with low mood (71–73) and anhedonia (74–76). Influence of fluoxetine treatment on activity in brain areas in positive (77) and negative (78) affect’s regime differ (79). On Figure 1C, one more phenomenon is demonstrated: circuits underlying constructs diagnostic for MDD are not identical. From comparison of these two simplified circuits for low mood and anhedonia, one can draw a conclusion that some regions are involved in the low mood but not in anhedonia and vice versa. Furthermore, there are regions such as the amygdala that are either up- or down-regulated in MDD, depending on which cognitive construct is present at the moment.

The circuits underlying constructs are overlapping and interacting; however, it seems that – as demonstrated on the example of fluoxetine – pharmacology targets specific constructs rather than the whole disorder. Interestingly, the same drugs are used in mental disorders sharing common cognitive constructs. For example, sertraline is used in the treatment of MDD, obsessive-compulsive disorder, panic disorder, anxiety disorders, post-traumatic stress disorder (PTSD), social phobia, and premenstrual dysphoric disorder, all of them involving fear (80).

## Modeling MDD

### Neural mass models and attractor landscapes

So far, psychiatric disorders have not been properly conceptualized in the language of computational neuroscience (81–83). Early research in this field was centered on reinforcement learning models which describe behavior as taking actions which maximize predicted rewards (84). Since DA is believed to be involved in prediction (85, 86), mostly the disorders linked to DA such as schizophrenia were modeled with use of the reinforcement learning (87).

However, since both calculating the odds for possible rewards and taking decisions on the basis of that calculation do not directly correspond to the neuronal activity and physiology of the brain, models based on reinforcement learning are a poor choice when it comes to neuroimaging-based biomarkers for mental disorders. In the last decade, comparing structural and functional connectivity in brain networks in health, in disease, in terms of graph theoretic measures, such as small-worldness (88) or modularity, (89) became a popular research direction (90). These measures have led to multiple interesting results upon the global properties of brain networks in cognitive disorders (91–93) including MDD (94, 95). However, these measures only take undirected connectivity between brain regions into account. The assumption of undirected connectivity yields a conclusion that for every pair of brain regions A and B, once treatment procedure targets region A, it has the same impact on region B, as if one would target region B with the same treatment and measure the change in activity in region A – which is, in general, an unrealistic assumption. Therefore, graph theoretic measures do not extensively incorporate the information that can be rendered from the neuroimaging data and that is of primary importance for assisting diagnosis and treatment in cognitive disorders.

Recently, the concept of attractor networks was proposed, as a tool that might explain cognitive disabilities while corresponding to the neural dynamics in the brain. An attractor network is a network of nodes, often recurrently connected, whose dynamics settle to a pattern stable in time: the so-called attractor state. Analysis of the distribution of attractor states and their basins of attraction, a so-called attractor landscape, was effected on a microscale so far. At the microscale, single neurons are the nodes in the network, and stable firing patterns of those neurons constitute an attractor state (96). This approach is present in contemporary computational neuroscience, e.g., in the models of activity in olfactory (97) and auditory (98) cortices in rodents as well as hippocampal grid cells in humans (99). This concept has also been broadly used in psychiatry. In example, the PFC has been modeled as attractor network in order to explain the deficit in short term memory in schizophrenia (100) and compulsions in obsessive-compulsive disorder (101). Up until now, it is unclear how these models translate to patients because neither the invasive measurements of a single-neuron activity necessary to validate the attractor network models are possible, nor do non-invasive methods have the appropriate resolution.

**Figure 2:**
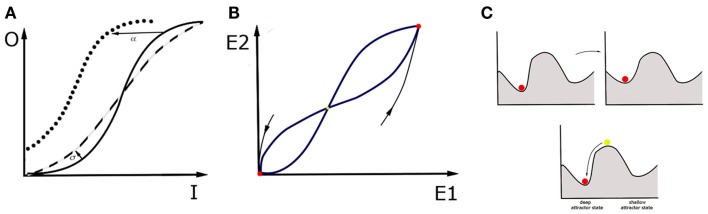
**Wilson–Cowan model and a “ball” metaphor**. The activity of a single brain area within the network is a consequence of the synaptic inputs from other areas, the modulatory tone generated by diffuse projections, and the recurrent connectivity within the brain area itself. The activity reflects a specific balance between excitation and inhibition within the area. For simplicity, we describe the activity by one variable, *E*, for which the following equation holds: $\tau \frac{\mathrm{dE}}{\mathrm{dt}}=-E+f\left(\alpha E+\beta I+\gamma M\right)$. The first term on the right tells us that in the absence of any drive (provided by the second term), the activity decays to zero with time scale τ. The second term incorporates the contribution of recurrent connectivity via *E* itself, input from other areas, represented by *I*, and the level of neuromodulation, represented by *M*. Each of these contributions are weighted by factors: α, β, and γ respectively. When the second term is positive, it increases the level of activity. The function *f* is a response function that translates the sum of activities into a driving term, and is typically sigmoidal (106): $f\left(x\right)=\frac{Ax^{2}}{x^{2}+\sigma^{2}}$. In this form, *A* is the maximum that *f* can reach for large *x* values, and σ is the value for which *f* is equal to half its maximum value. In addition, it also specifies how steeply *f* increases with *x*, a quantity that is also referred to as the gain factor. Note that this expression only holds for positive *x* values, *f* is zero when *x* is negative. This model has a range of parameters, which is important because each of them can be linked to specific physiological processes and changes in circuit structure. For instance, an increased β represents a stronger synaptic projection, whereas an increased α represents stronger recurrent synapses. An increased *M* reflects the effect of neuromodulators that increase the level of depolarization in the cells, and hence the baseline firing rate; γ reflects the sensitivity to neuromodulators of cells and circuits. The value of σ can be interpreted as a change in gain. **(A)** In a given region, the sigmoidal input-output (I-O) relationship has three regimes. For small input y<<σ, it increases rapidly. For large inputs, y>>σ, it saturates. For values in between, it connects these regimes linearly. If the σ value, and thus excitability of the region, grows (dashed line), the I-O function is steeper than in the control case (solid line). If the region gets stronger recurrent connectivity, input from other regions or neuromodulation, so that the α, β, γ values grow respectively, I-O function shifts to the left (dotted line). **(B)** In an example of two interconnected regions, *E*_1_ and *E*_2_, this dynamical system has three fixed points that are candidates for attractor states. In this example, two of them are stable (red). For a given attractor, setting activities *E*_1_, *E*_2_ to arbitrary initial values within the basin of attraction will make the system move on toward this attractor. The third fixed point is unstable (yellow), which means that every small perturbation from this state makes the system fall into one of the basins of attraction, and thus end up in one of two attractor states. **(C)** One may picture attractor states with the ball metaphor. Disease can be represented in two ways. It can mean a change in the landscape of basins of attraction: some attractor states change position and even if the patient occupies the original attractor throughout the process, their brain state gradually changes the attractor state that they occupy. This can be achieved by changing shape of I-O function with use of parameters σ and α, β, γ or changing of relaxation time constants τ. However, it can also mean that, in a result of intrinsic noise in the brain or in response to a particular external input, the brain state in the patient is triggered to switch to another “wrong” basin of attraction. The noisy behavior of the network is not captured by the basic version of Wilson–Cowan equations, but incorporating noise in and therefore also a stochastic driving force is also possible. An attractor is a network state where the levels of activity do not change anymore, hence *E* is constant. Mathematically, this means that *E* does not change over time, hence that its value is given by setting the right hand side of equation (1) to zero, which yields E=f(αE+βI+γM), hence *f* gives the steady state values, hence increases in the factors α, β, and γ immediately increase the *E* value. It is important to realize that this is an equation from which *E* needs to be found. In the preceding, we focused on a single variable *E*, but in a network there is at least one variable for each brain area involved. For multiple brain regions involved, which is true in MDD, $\tau_{i}\frac{dE_{i}}{\mathrm{dt}}=-E_{i}+f_{i}\left(\sum_{j}J_{\mathrm{ij}}E_{j}+\gamma_{i}M_{i}+I_{\text{stim},i}\right)$. Here *i* represents the index of the brain area and *j* is the index of brain areas that provide input. Most parameters now have an index *i*, because their value depends on the area they represent. We have also included a stimulation current, which represents the effects of electric or magnetic stimulation. Within this framework, the effects of treatments can be captured. On one hand, treatments can reshape the attractor landscape. For instance, pharmacological manipulations can either change the level of neuromodulation or the sensitivity of the circuit to neuromodulators. This would lead to the homeostatic regulation of the coupling coefficients *J*_*ij*_, and σ, and, subsequently, to the change in the map of attractors. On the other hand, a single electrical stimulation, such as ECT session, could change the attractor, offering temporary relief; but if the new attractor is not stable, the brain network could return to the old attractor over time. A sequence of electrical stimulation would also affect *J*_*ij*_ and thus change which attractors are possible and how stable they are. Taken together, electrical stimulation has the advantage that its effect is local and can be tuned to alter/correct a specific *J*_*ij*_ value.

How about the macroscale? It is now believed that the fMRI research can provide the insight necessary to understand cognitive constructs (102, 103). But is the concept of attractors also applicable for this sort of data? Here, we propose a conceptual advance to apply mathematical modeling directly to patients. This proposal involves looking at the large-scale neural circuits in order to perform attractor landscape analysis on the macroscale. Mind that brain circuits are networks of interacting nodes, and therefore can be represented and analyzed as dynamical systems, in a similar fashion as networks of single neurons. As opposed to microscale, at the macroscale whole brain areas account for the nodes in the network, and attractor states are stable activity patterns across all nodes within the network. For example, in case of the fMRI data, the overall activity in a region of interest can be expressed as the summation over activity of all voxels within that region. This data is very convenient for neural mass models when it comes to modeling cognitive architectures (104). The principal idea of neural mass models is setting the density of neurons to the continuum limit in modeling the activity of large neural populations. This assumption of spatially continuous neural networks thus allows for analytical treatment of such global variables as firing rate in space and time. An example is the classic Wilson–Cowan mean-field model (105). In this model, the activity of neuronal populations (or brain regions) is represented by dynamical variables. Figure 2 presents a simplified version of the model where spatial patterns of spiking activity are replaced by one dynamical variable. In the model, effectively connected neuronal populations, representing brain regions, interact and are additionally tuned by neuromodulators. Such dynamical systems have a number of stable attractors, and therefore a number of basins of attraction. The possibility is that in MDD patients, the shape of the attractor landscape for a particular cognitive construct is different than in healthy controls. However, it can also be that they occupy a “wrong” attractor state (106).

### Treatments in the context of dynamical systems

All of the available treatments affect the dynamics of large-scale networks and therefore also the attractor landscapes (108–110). Therefore, with use of the Wilson–Cowan model, one can then investigate the landscape of basins of attraction in response to the treatment procedures. Antidepressant drugs can reshape the attractor landscape in multiple ways: they can lower the hills of the landscape around the current state of the patient or make the current attractor state shallower in order to facilitate escaping from the local minimum (Figure 2C, upper). The drugs can potentially also modify background neuronal noise, which in turn may affect the probability of occupying different attractor states (111). On the other hand, stimulation methods that regulate the neural dynamics directly, such as rTMS, ECT, and DBS can influence the state of the patient by providing a brief pulse to the brain network in the patient and thus allowing the brain network to leave the “wrong” attractor state immediately (Figure 2C, lower). Interestingly, in the treatment-resistant depression, electrical stimulation through ECT and DBS prove to be highly effective (112, 113), which means that, under some circumstances, they perform better than drugs, or even than the cognitive therapy which targets the cognitive constructs directly. This provides some hint suggesting that looking at clinical symptoms of MDD through the prism of neuronal circuits, and targeting treatments at those circuits might be more beneficial than any other treatment, including, paradoxically, even the behavioral treatment centered at specific cognitive traits in MDD.

## Circuit to Construct Mapping

### What is CCM

Every patient has a different, individual attractor landscape. This landscape reflects such personal traits as the size of the brain regions involved in MDD, functional connectivity within DMN and CEN, baseline concentrations of monoamines, and all the other endogenous chemicals that influence the excitation-inhibition balance in the brain. During rest, DMN and other RSNs are active and the patient occupies stable attractors in their attractor landscapes. On the contrary, during solving cognitive tasks, subnetworks of CEN come to play (depending on the nature of the task) and the brain state jumps to one of its (most probably, also stable) attractors. We predict that a disturbance of the attractor landscapes within the DMN should account for the cognitive constructs involving affective components of MDD, whereas disturbance of the attractor landscapes within cognition-related RSNs (such as fronto-parietal network) and within the CEN should be responsible for the cognitive constructs involving executive functions.

**Figure 3:**
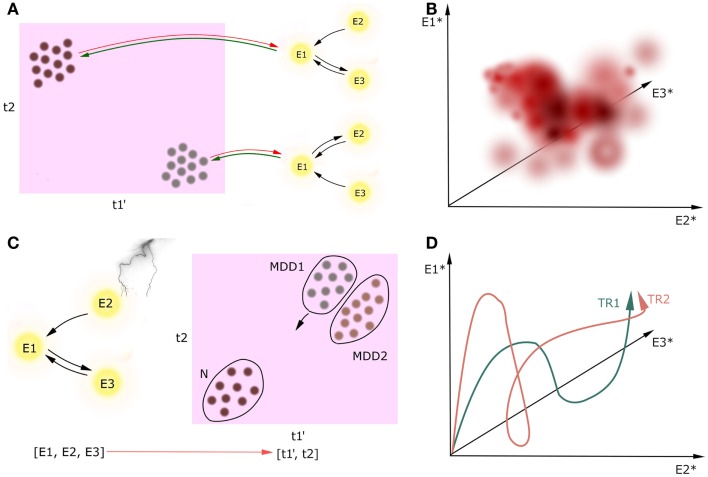
**Circuit to construct mapping**. Causality between activity in underlying network (nodes E1, E2, E3) and the multidimensional construct space (t1, t2) can go two ways, but we are only interested in neural circuitry as the cause and cognitive constructs as the effect [**(A)**, green lines]. Behavioral learning and neuroplasticity can give the backward direction of causality [**(A)**, red lines]; however, we do not cover this issue in this paper. We refer to cognitive constructs as t_i_ because the preliminary step of the CCM includes determining the full list of the involved constructs which can be broader than the list of the DSM-5 diagnostic criteria. Prime denotes endophenotype, dot denotes a patient, asterisk denotes attractor state. Firstly, circuitries involved in these constructs should be linked on the basis of extensive observational research on a large cohort of patients **(A)**. Secondly, we can plot how these attractors map onto cognitive constructs with temperature maps **(B)**. In this example, we plot the value of one continuous construct (which can represent, i.e., level of mood) in a three-dimensional space spanned by the attractors of the underlying three-node network. Thirdly, one can track the current state of one patient both in the multidimensional construct space during the treatment [**(C)**, a scatter plot in case we want to track multiple patients at a time]. Distribution of patients in this space may reveal subtypes of MDD (MDD1, MDD2). Moreover, when the network manipulation with treatment is sufficient, it can trigger the patient’s brain state toward a new attractor in some of the construct-related dimensions, and in a result the patient flows to another point in the construct space. Since we will create these maps on the data from the limited number of patients in the cohort, this temperature maps will not span the whole volume of possibilities. Lastly, one may investigate how the treatments TR_i_ affect the attractors of the underlying networks **(D)**. In this case, we have three nodes in the underlying networks, which means that the attractors of this system will be points in a three-dimensional attractor space.

But how do these attractors map onto cognition? Let us consider a brain network consisting of interconnected nodes described by their activities, either in resting state or in some cognitive process (Figure 3). While looking for causal interactions between neuronal circuitry and behavioral outcome, one should perform a mapping from a multidimensional space spanned by patterns of neuronal activity (namely, attractors of the neuronal networks) onto a multidimensional space spanned by the cognitive constructs. This is what we called the CCM approach. The direction of causal inference in CCM goes from circuitries toward behavior because the CCM approach is designed for better treatment, which should ultimately target the diagnostic cognitive constructs in MDD. Therefore, it is essential for the constructs to be compact, but the underlying circuits can be complex as is necessary.

The CCM approach involves performing this mapping with use of joint imaging and psychometric methods on large clinical datasets. Once we identify the circuits underlying single cognitive dimensions of MDD, we can perturb this construct-related circuits in a single patient with treatments, affecting the neuronal dynamics, and tracking both the resulting position in cognitive construct space and the dynamical properties in the construct-related circuits.

### Execution of CCM

Execution of CCM is a multistep process. The preliminary step is to determine an extensive list of constructs involved in MDD. Since the classic diagnostic tools are questionnaires and experimental tasks, this analysis would run through a number of various variables, grouping them into dimensions, with a subsequent sanity check if the outcome constructs have a consistent content. The list of constructs determined in this protocol can be longer than the list of the DSM-5 criteria, thus we call the constructs with anonymous *t_i_* in the Figure 3A. Furthermore, some constructs may be heritable and thus fulfill the definition of endophenotypes, which is especially relevant for executive functions (114), whereas other constructs such as recurrent thoughts of death are not likely to be heritable. However, this analysis will not reveal whether a given construct is heritable or not.

The second step is to find neuronal mechanisms of each of the obtained constructs. For every single construct, one should start the procedure from the first order analysis: investigating patterns of activation and effective connectivity in a cohort of patients exhibiting that construct (and, of course, a cohort of controls), in order to identify the underlying neuronal network and to build a corresponding dynamical system (Figure 3A). Using Pearl’s definition of causality, for the effect size large enough we can determine causal effects on the basis of this observational study.

If this first level analysis does not identify unique circuitry, there can be multiple interacting circuitries involved in the construct. In that case, one should perform a second order analysis. For instance, one can perform repeated diagnostic evaluation and repeated fMRI imaging assessment longitudinally within the same patient. Then, using autoregressive models in order to analyze the time course of the construct and correlating these independent components with neuroimaging data should reveal independent components in the circuitry underlying this construct.

We predict that positive correlations between revealed cognitive constructs across patients are inevitable, which should be reflected in overlaps between circuits underlying the constructs. We can also analyze how the attractors of the dynamical systems map onto cognitive constructs using temperature maps (Figure 3B). Since we will create these maps on the data from the limited number of patients in the cohort, this temperature maps will not span the whole volume of possibilities.

The third step is building the dynamical models representing the identified circuitries underlying cognitive constructs. The proposed Wilson–Cowan model can be applied to any clinical data that reveals the distribution of activity in the brain over time (115), in particular to blood oxygen level dependent (BOLD) signal in fMRI (116) or EMG/EEG data (117). Wilson–Cowan model has some similarities to the dynamical causal modeling (DCM), a well established method for extracting effective connectivity for both fMRI and EEG/EMG data (118–124), in a sense that it describes the neuronal communication between brain regions in terms of ordinary differential equations. The major difference is that – in both classical (119) and recent stochastic version of DCM for fMRI data (125) – there is an assumption of linear transfer functions, whereas it is known that large neuronal populations exhibit sigmoidal rather than linear response to the external inputs (106), which is incorporated in the Wilson–Cowan equations (126).

In this procedure, a single patient in a cohort is just an object to the explanatory science. However, once the circuitries underlying cognitive constructs involved in MDD are determined, the patient may become a subject in a case study, and receive a personalized treatment. Investigation of the trajectory of the particular patient in the construct space in response to changes in the circuit activity caused by treatments (Figure 3C) might not only provide new biomarkers for MDD and better insight into the mechanisms of treatments, but also answer the question of how to predict resilience to treatment. This research may also elucidate factors that determine whether a treatment is effective to a particular group of patients. Furthermore, this analysis might help to address the question if the mental disorders of interest, e.g., MDD, are homogenous or split into subtypes on the basis of the patient trajectories in the construct space. Lastly, one may investigate how the treatments TR_i_ in the given patient affect the attractors of the underlying networks (Figure 3D).

### Benefits of CCM

**Figure 4:**
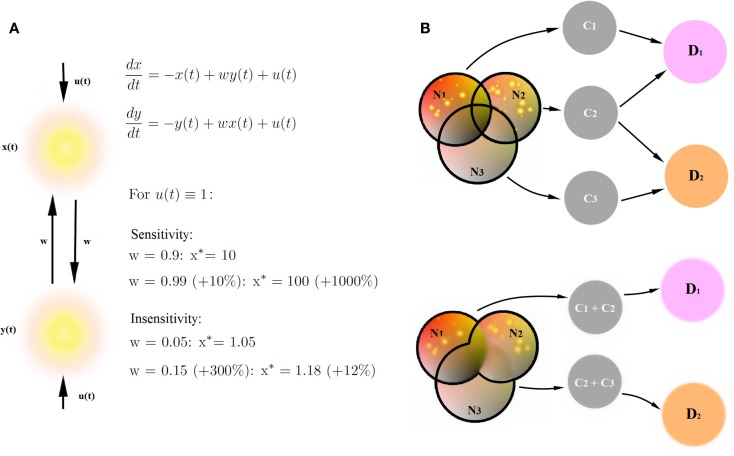
**Benefits of CCM**. **(A)** Stable connectivity patterns and connectivity strengths are two sides of the same coin. Let us assume that our sigmoidal transfer function can be approximated as a simple linear function. We provide an example of a network sensitive to changes in connectivity strengths (a small change in connection weights yields an huge change in the value of stable activation patterns) and an example of a network insensitive to changes in connectivity strengths (a huge change in the connectivity strengths yields a small change in stable activation patterns). Therefore, the description of networks by means of dynamical systems provides more versatile description than connectivity strengths in the networks or stable connectivity patterns alone. **(B)** Decomposition of psychiatric disorders into a number of diagnostic traits helps causal inference in the diagnostic process. Networks N_i_ underlie cognitive constructs C_i_ diagnostic to psychiatric disorders D_i_. Disorders D_1_ and D_2_ share a common cognitive construct C_2_, but involve also disorder-specific diagnostic constructs C_1_ and C_3_. Let us assume that the joint posterior distribution for every disorder D_i_ factorizes into posterior probability distributions for single diagnostic constructs. Let us further assume that we find the same, specific pathologies in networks N_1_ and N_2_. If we can decompose the D_1_-related network into a sum of networks N_1_ and N_2_ underlying single diagnostic constructs C_1_ and C_2_ (upper panel), we collect more evidence for the disorder D_1_ (pathologies in both networks linked to diagnostic constructs) than for the disorder D_2_ (pathology in one out of two networks linked to diagnostic constructs). On the other hand, if we are not able to decompose D_1_ and D_2_-related networks into networks underlying single diagnostic categories (lower panel), the amount for evidence in favor of both disorders is the same because both networks (N_1_ + N_2_) underlying disorder D_1_ and (N_2_ + N_3_) underlying disorder D_2_ are pathological.

Circuit to construct mapping brings three new qualities to the table. Firstly, treating networks as dynamical systems allows one to extract and to characterize global properties of the networks involved in cognitive constructs in a comprehensive and versatile way. So far, research in human imaging was focused on finding particular areas involved in cognitive tasks by virtue of stable activation patterns, or investigating context-dependent strength of connectivity between particular areas. These are two out of many viewpoints which one can take in order to characterize large-scale brain networks. In fact, these are the two sides of the same coin: the distribution of activation patterns in a network is a global property emerging from behavior of the underlying dynamical system specified through the connection strengths between areas. Whether the activity patterns are more informative than the connectivity strengths, depends on the circumstances. In Figure 4A, we present a toy example. Let us assume that, in the simplest case, our sigmoidal transfer function can be approximated as a linear function. For some combinations of inputs to the network and connection weights, a small change in connection weights (by 10%) yields an enormous change in the value of stable activation patterns (by 1000%, upper panel). For other combinations of weights and inputs, even huge change in the connectivity strengths (by 300%) yields a small change in stable activation patterns (by 10%). As a consequence, whether activity patterns in the networks are sensitive to changes in connectivity strengths depends on the tuning in the network, for instance on the balance between connectivity weights in the network and external conditions such as experimental inputs. Therefore, since the dynamical systems incorporate both connectivity (as the cause) and about stable activity patterns (as the effect), they integrate the two sorts of information about the circuits into one framework.

Secondly, the decomposition of psychiatric disorders into a number of diagnostic traits allows for fundamental explanatory research in psychiatry, and therefore also for new, neuroimaging-based biomarkers for cognitive disorders. In terms of causal modeling, gathering clusters of traits into big cognitive paradigms such as psychiatric disorders can be misleading, given that the disorders strongly overlap in terms of diagnostic criteria. A simple example is provided in Figure 4B. In this example, overlapping networks N_i_ underlie cognitive constructs C_i_, which are diagnostic to psychiatric disorders D_i_. Disorders D_1_ and D_2_ share a common cognitive construct C_2_, but involve also disorder-specific diagnostic constructs C_1_ and C_3_. In this toy example, let us assume that the prior probabilities of cognitive constructs C_i_ are equal and that likelihood of the pathologies in networks N_i_ given constructs C_i_ are the same. Let us further assume that in our patient, we find the same, specific pathologies in networks N_1_ and N_2_. If we can decompose the D_1_- related network into a sum of networks N_1_ and N_2_ underlying single diagnostic constructs C_1_ and C_2_ (Figure 4B, upper panel), we can perform statistical inference, linking specific changes in N_1_ and N_2_ with constructs C_1_ and C_2_, respectively, and collecting evidence behind the hypothesis that the patient is a subject to the disorder D_1_. Since C_2_ is also a construct diagnostic to the disorder D_2_, we also collect some evidence behind the hypothesis that the patient suffers from the disorder D_2_. However, assuming that the joint posterior distribution for every disorder D_i_ factorizes into posterior probability distributions for single diagnostic constructs, we collect more evidence for the disorder D_1_ than for the disorder D_2_.

On the other hand, if we are not able to decompose D_1_ and D_2_-related networks into networks underlying single diagnostic categories (Figure 4B, lower panel), the amount for evidence in favor of both disorders is the same because both networks (N_1_ + N_2_) underlying disorder D_1_ and (N_2_ + N_3_) underlying disorder D_2_ are pathological, and we are not able to extract any disorder-specific subnetworks which would provide any further evidence in favor of one of the disorders. Therefore, decomposing mental disorders into single diagnostic constructs and linking construct-specific circuits is of primary importance for explanatory models in psychiatry.

Thirdly, CCM as a modeling procedure that projects neuronal dynamics straight into behavioral dimensions of MDD, could not only serve as explanatory model when applied to a large cohort of patients, but also enhance the current treatment selection for individual patients and make a step toward the personalized medicine. In order to perform explanatory research “in Pearl’s sense,” we need to use neuroimaging along with behavioral data from a large cohort of patients because, in order to reveal the circuitries underlying MDD-related cognitive constructs, we need to find systematic differences in circuit dynamics that result in systematic differences in behavior. But once this explanatory research is done and the circuitries underlying cognitive dimensions of MDD are defined, zooming into the circuit dynamics and its development under treatment in a particular patient would allow for the personalized interventions.

## Limitations of CCM Approach

### Plasticity and neurodegeneration

So far, sensory systems are best characterized in terms of underlying circuitries. However, events in sensory systems happen on a millisecond to second timescale whereas the evolution of psychiatric disorders is a few orders of magnitude slower and therefore might be much more complex. MDD may result from traumatic experience or emerge without a particular inducing event, but in any case the process of falling into a depressive episode lasts for weeks, as opposed to perceptual learning which takes only seconds. Also, some treatment procedures are long lasting, i.e., MDD pharmacotherapy is primarily monoamine based and typically requires intake for 3–4 weeks prior to symptomatic improvement (with the exception of ketamine). This time course is a major impediment to modeling MDD because imbalance in mood may arise not only on top of changes in neurotransmitter concentrations, but also result from other processes such as structural plasticity and neurodegeneration (127). The mechanisms underlying these two processes are not fully understood, and, in the case of structural plasticity, is difficult to investigate in a living human brain. Neural mass models can only serve to compare between different stages of the disorder in an individual, and between different individuals at the same stage, yet does not provide a framework that demonstrates real-time evolution of MDD.

### Heterogeneity

MDD is a heterogenous disorder. The diagnostic criteria are still evolving, and the recently published DSM-5 diagnostic criteria for MDD allow for a variety of diagnostic combinations of cognitive constructs. Is there a plethora of different MDD types, or rather one prevalent state of mind that manifests itself in various ways depending on the patient? This remains an open question. Furthermore, in the literature, there is often no clear distinction between patients who experience a first depressive episode and those who suffer from recurrent depression whereas, as neurodegeneration proceeds and the severity of symptoms elevates, the course of the disease plays the crucial role in the treatment procedure. This also provides a hindrance to the modeling procedures since the information about the stage of the disease is often missing from databases.

**Figure 5:**
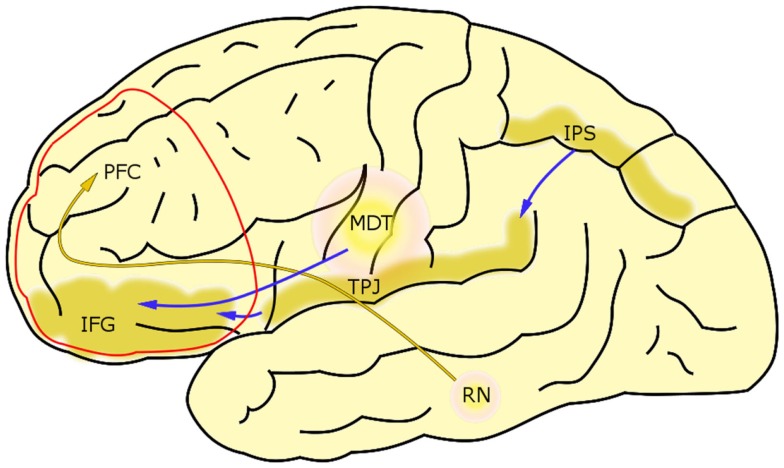
**Attention as an example of a construct with multiple neural mechanisms underneath**. Maintaining attention can be disrupted by at least two distinct mechanisms: (1) Oversensitivity of the ventral attention network. Imaging studies revealed two systems managing attention in humans. On one hand, we have dorsal attention system, consisting of frontal eye fields (FEF) and intraparietal sulcus (IPS), controlling voluntary deployment of attention (top-down control). On the other hand, we have a right-lateralized ventral attention network (VAN), responsible for orienting attention toward sensory stimuli. It involves temporoparietal junction (TPJ), intraparietal sulcus (IPS) in the parietal cortex, and inferior frontal gyrus (IFG). IFG, as a part of orbitofrontal cortex, receives a strong excitatory input from medial dorsal thalamus (MDT). Since MDT is overactive in MDD, this effect can make ventral attention network oversensitive to stimuli, and as a result holding attention on salient stimuli becomes difficult to the patient. (2) Diminished communication through coherence in the prefrontal cortex. Serotonin produced in the raphe nucleus (RN) modulates gamma oscillations in the prefrontal cortex (PFC), most probably by acting on fast-spiking interneurons expressing serotonin 5-HT_2_ and 5-HT_6_ receptors. Gamma oscillations play a key role in higher cognitive processes, including attention and working memory. Since serotonergic input to the prefrontal cortex is known to be diminished in MDD, the decrease in gamma power may account for the effect of distractibility in MDD. Both of the above mechanisms lead to a decrease in inhibition within the prefrontal cortex, which might explain why the attention, managed in the PFC, both can be disrupted in a result of hyperactivity of the medial dorsal thalamus and hypoactivity of the raphe nucleus.

Furthermore, complexity of MDD might project also to strongly overlapping construct-related circuits. In example, it was found that the same brain area may host different circuits, which, when activated, have opposing effects on anxiety (128). Furthermore, fMRI studies reveal anticorrelated networks to be activated during cognitive tasks (129). This is circumstantial evidence that multiple distinct circuits can underlie single cognitive constructs (Figure 1C). Furthermore, the same constructs can arise from different mechanisms. In Figure 5, we discuss impairment in maintaining attention as an exemplary construct that may develop in the PFC of the MDD patients from distinct processes.

### Application of treatments to the CCM

Some of the possible applications of CCM such as DBS and ECT require invasive methods that cannot be used in humans on a daily basis, and thus require rodent models. Rodent models of MDD are a well explored discipline. However, whether rodent models in mental disorders are fully translational remains unclear, which presents another difficulty for modeling studies. Whereas anhedonia, weight loss and gain, hypersomnia, or psychomotor retardation can be measured in a rodent, some other constructs such as the presence of recurrent thoughts of death, have no equivalent in rodents. On the other hand, modeling that requires invasive techniques such as electrophysiology cannot be ethically introduced into living human brains except under certain prescribed neurosurgical situations. However, the TMS-, pharmacotherapy- and neurofeedback-related CCM approach constitutes an adjunct to rodent models and, as a non-invasive method, it is applicable to patients. Among the emerging treatment methods, neurofeedback seems to be a promising therapeutic procedure for CCM. This method is known to change connectivity in the functional networks (130, 131), but its mechanisms of action are not yet known. Yet the concept of guided self-modulation in a patient in absence of any third-party tools such as electric current or drugs is tempting. However, CCM can also be paired with all the other treatment procedures.

What can be a hindrance in application of the pharmacotherapy-related CCM is that it is difficult to target a given construct with a particular drug because MDD drugs act on monoamine receptors, which are ubiquitous in the brain and present in multiple circuits at a time (Figure 1C). Furthermore, some brain regions are hubs that are affected in many constructs thus, targeting these nodes with any form of treatment will have broad consequences for the global brain state. For example, the ventral medial PFC is a major hub in the limbic system known to be involved in low mood (72), anhedonia (75), feelings of worthlessness (132), and diminished working memory (133) in MDD. However, the idea is to provide the online readout for the dynamics of all the involved circuits at a time. Due to this approach, the clinician may first apply a specific treatment in order to target a desired cognitive construct, and then observe how the other construct-related circuits evolve along with the targeted one.

### Temporal dynamics in the resting state

Circumstantial evidence suggests that in some aspects, MDD might require deeper insight into activity of neural networks than the afforded by global patterns of activity in the populations of brain regions as obtained from fMRI studies. For example, the DBS has different remission rates depending on the temporal characteristic of the applied current. As it was recently demonstrated that in the Parkinson’s disease, temporally irregular DBS is more effective than oscillatory stimulation (134). This effect suggests that in addition to the modulatory effect on E-I balance, electrical stimulation can change the communication between the targeted region and its efferents by affecting communication through coherence (135). This means that the fMRI data, as they are lacking the temporal characteristics in the brain activity, might give an incomplete information about mechanisms of MDD. However, CCM is still a substantial progress for the therapy and treatment in mental disorders, and gives a first insight into the circuits involved in the disorder that opens possibilities for further, more in depth research.

### Effective connectivity in EEG/EMG and fMRI research

So far, there are papers whose authors use Ising models in order to provide a global description of network properties (as a number of so-called patterns stored in the network (136). However, Ising models are defined only for undirected networks and, in order to use full potential of the CCM, this approach needs a step further by making connectivity directional. In fMRI research, parcelation of the brain into regions is quite successful (137; Oort, in preparation); however, determining connectivity strengths between the nodes is harder because of the limited amount of the temporal information in the fMRI data. So far, the only widely used inference procedure for effective connectivity on the basis of fMRI data is the aforementioned DCM; however, it is only applicable for very small networks 3–4 nodes, requires predefinition of a number of parameters and of network nodes, and in addition to that, as an inference procedure, encounters some critics in the field (138). Since region definition in causality for fMRI is extremely important (Bielczyk et al., in preparation), there is an urge for new, more data driven methods for approaching effective connectivity in these datasets.

In the field of EEG/EMG on the contrary, the problem of causality is orthogonal to the fMRI field: the DCM procedure is quite successful in finding effective connectivity between the nodes of the network, however the optimal method for defining the nodes as sources of the potentials recorded on the scalp is still an open problem. Three popular approaches are dipole modeling, dynamic imaging of coherent sources and frequency-domain minimum current estimation (139). These methods successfully identify the main sources of oscillations in the brain volume, however there is a room for improvement in terms of the spatial resolution of reconstructed sources.

## Concluding Remarks

As proposed by RDoC initiative, symptoms diagnostic for psychiatric disorders should be interpreted as psychopathological constructs, which need to be investigated, diagnosed, and treated independently. The CCM approach addresses this demand, and provides with a new outlook at clinical treatments in mental disorders. Namely, the treatments not only regulate levels of neuromodulatory substances but also change the dynamical state of the brain by regulating excitation-inhibition balance across brain circuits, which can be tracked with neuroimaging. This change in dynamics may be achieved in two ways: by inducing the structural and functional plasticity that changes the functional connectivity in the circuit (through drugs), or by providing stimulation/inhibition to discrete circuit node (s) and therefore changing the global balance in the brain (through electrical stimulation).

In this work, we underscore the potential of computational modeling in psychiatry as a tool to unravel mechanisms underlying the diagnostic symptoms, to cluster diagnostic cohorts and to customize approach to clinical populations in psychiatry. In addition to this, we anticipate that in the near future, new, personalized treatment methods based on non-invasive regulation of specific neuronal populations’ activity with gene therapy may be possible. This approach is still in its infancy and remains to be clinically validated. However, gene therapy up-regulation of p11 protein in the rodent nucleus accumbens proved to cause a reversal of an anhedonic phenotype (140).

Due to our assumptions, diagnostic symptoms of MDD are caused by (mal)behavior of the underlying neuronal circuits. Therefore, we suggest that clinical groups homogenous in the circuit dynamics should also be responsive to similar treatments. Conducting the diagnosis in terms of circuit defects based on the construct domain will then ensure the clinical groups are clustered, and represent more homogenous groups. Furthermore, comparison of depressed patients and healthy controls in the construct space may assist in the investigation if MDD is a single disorder (and diagnostic category) or whether it should be split into diagnostic subtypes. It may also reveal cognitive and neuronal signatures of the phenomena of treatment-resistance. Tracking patient’s position in the construct space in response to stimulation/inhibition on one hand, and the evolution of relevant attractor landscapes on the other hand, may provide new insight into the nature of treatments and help to create personalized medicine.

## Author Contributions

Collecting materials: NB. Drafting of the manuscript: NB. Critical revision of the manuscript, clinical part: JG, JB. Critical revision of the manuscript, computational part: PT.

## Conflict of Interest Statement

The authors declare that the research was conducted in the absence of any commercial or financial relationships that could be construed as a potential conflict of interest.
